# Artificial Intelligence: Is It Armageddon for Breast Radiologists?

**DOI:** 10.7759/cureus.8923

**Published:** 2020-06-30

**Authors:** Lawman Chiwome, Onosetale M Okojie, A. K. M. Jamiur Rahman, Faheem Javed, Pousettef Hamid

**Affiliations:** 1 General Internal Medicine, University Hospitals of Morecambe Bay NHS Foundation Trust, Lancaster, GBR; 2 Family Medicine, California Institute of Behavioral Neurosciences and Psychology, Fairfield, USA; 3 Internal Medicine, California Institute of Behavioral Neurosciences and Psychology, Fairfield, USA; 4 Anaesthesia, California Institute of Behavioral Neurosciences and Psychology, Fairfield, USA; 5 Neurology, California Institute of Behavioral Neurosciences and Psychology, Fairfield, USA

**Keywords:** artificial intelligence, radiology, breast cancer, mammogram

## Abstract

Artificial Intelligence (AI) has taken radiology by storm, in particular, mammogram interpretation, and we have seen a recent surge in the number of publications on potential uses of AI in breast radiology. Breast cancer exerts a lot of burden on the National Health Service (NHS) and is the second most common cancer in the UK as of 2018. New cases of breast cancer have been on the rise in the past decade, while the survival rate has been improving. The NHS breast cancer screening program led to an improvement in survival rate. The expansion of the screening program led to more mammograms, thereby putting more work on the hands of radiologists, and the issue of double reading further worsens the workload. The introduction of computer-aided detection (CAD) systems to help radiologists was found not to have the expected outcome of improving the performance of readers. Unreliability of CAD systems has led to the explosion of studies and development of applications with the potential use in breast imaging. The purported success recorded with the use of machine learning in breast radiology has led to people postulating ideas that AI will replace breast radiologists. Of course, AI has many applications and potential uses in radiology, but will it replace radiologists? We reviewed many articles on the use of AI in breast radiology to give future radiologists and radiologists full information on this topic. This article focuses on explaining the basic principles and terminology of AI in radiology, potential uses, and limitations of AI in radiology. We have also analysed articles and answered the question of whether AI will replace radiologists.

## Introduction and background

Women in the UK have one in eight chances of developing cancer of the breast during their lives [1]. According to the official statistics of 2018, breast cancer is the second most common cancer diagnosed in the UK, with 47,476 recorded cases. It has been the most frequently diagnosed cancer in England since 1996 [2,3]. The incidence rate increased from 2008 to 2018 while the mortality rate decreased as shown in Table 1.

**Table 1:** Age-standardized mortality and incidence rates per 100,000 for breast cancer in females, England, 2008 and 2018 Statistics credit: Cancer registration statistics, England [3].

2008		2018	
Mortality rate	Incidence rate	Mortality rate	Incidence rate
39.9	165.5	34.1	167.7

This decreasing mortality contrasts to the increase in new cases, indicating that the number of patients surviving breast cancer has improved [3]. This is as a result of early detection of breast cancer through expanded mammography screening [3]. Mammography is the best method available to detect cancer of the breast before the lesions become clinically visible, and mortality is reduced by as much as 30% [4]. However, mammograms are complex, and the high numbers (1.79 million mammograms done in 2017-18 under the National Health Service [NHS] Breast Screening Program) of exams per reader can result in inaccurate diagnosis [1,5]. The incorrect diagnosis led to double reading of mammograms in the UK and Europe [6]. When using double reading, sensitivity of mammography is increased by 5%-15% when compared with single reading [6]. Computer-aided detection (CAD) systems introduced because radiologists missed about 25% of visible cancers on mammograms due to interpretation errors [7]. The reason for the introduction of CAD systems was to try and improve human detection performance [8]. However, recent studies show that CAD models have not had the expected impact, and there was no significant improvement in the diagnostic accuracy of mammography, or reduction in recall rates [9]. Advances in AI are promising to address the flaws of CAD systems [10]. A present-day breast radiologist and those aspiring to be radiologists should be aware of the fundamental principles of AI, its use, limitations, and what the future holds. The nitty-gritty of AI is not of much importance to radiologists. Still, they must know the technical jargon used by application developers to communicate with them and be prepared for the future efficiently.

## Review

Artificial intelligence

Artificial intelligence (AI) technology has existed for more than half a century and has become more and more sophisticated [11]. The first reports on AI use in radiology date back to 1963, but the flames of those years quickly dozed off [12]. A recent increase in amounts of electronic medical data and technological improvements brought with it a new vigour in AI applications [11-13]. AI is defined as a technology that could broadly mimic the intelligence of humans [11-13]. AI has two broad categories, namely, general AI and narrow AI [11-13]. General AI is a concept where machines can function and exhibit all the intellectual capabilities of humans such as reasoning, seeing and even hearing, whereas narrow AI is where technologies are only able to do specific tasks [11-13]. Narrow AI is what is achievable at this time, and general AI is still a pipe dream.​​​​​​

Machine Learning

The Idea of introducing technology in medicine started as a tool known as expert systems, and the goal was to make algorithms that would make decisions like physicians [13-15]. However, it was not possible to make an algorithm for all diseases given the different presentations and scenarios encountered in day-to-day medicine [13-15]. Machine learning (ML) then took over from expert systems in the 1990s [13-15]. ML is a component of AI, and it tries to replicate the learning part of human brains [13-15]. In ML, the algorithm learns from exposure to large and new data sets and improves with continuous exposure to data [13]. There is no need for explicit programming in ML, and this differentiates it from standard programming, which requires clear step-by-step instructions the program must take [14].

Categories of ML

ML can be divided into supervised, unsupervised and reinforcement types of learning [13-15]. In unsupervised learning, there is no labelled input data [13-15]. The algorithm is supposed to analyse the data, group the data, and produce an output. There is no feedback supplied [13-15]. In reinforcement learning, the algorithm analyses data, and it is either rewarded or punished depending on the accuracy of the output it produces (reinforcements) [13-15]. The machine learns how to act in a particular environment to maximize rewards [15]. Supervised learning is when the algorithm is provided with labelled data to learn from, and this is known as the training phase [15]. The algorithm is expected to find recurring patterns from the training data and be able to pair inputs to results. In radiology, this means the mammogram diagnosis suggested by AI has to match tissue diagnoses [13].

Artificial Neural Networks

Artificial neural networks (ANNs) are a component of ML that uses mathematical and statistical principles to analyse data [16]. These networks' development came after inspiration from the biologic nervous systems' way of processing information using a large number of highly interconnected neurons [16]. An ANN has one input layer and one output layer of neurons [13-16]. In between the input and output is one or more layers known as "hidden layers." A hidden segment consists of a set of neurons, with connections to all neurons in the previous and forward layers [13-16].

Deep Neural Network (Deep Learning)

**Figure 1:**
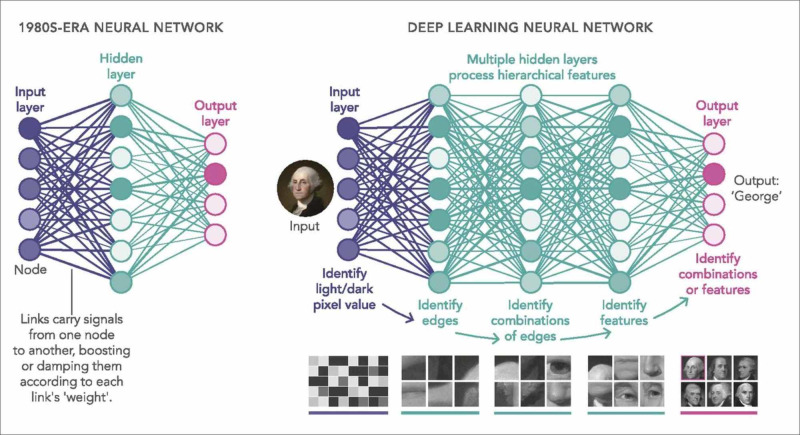
Simple neural network compared to the deep neural network Photo credit: Waldrop [17].

Deep learning (DL) is the AI concept used in image interpretation. A deep neural network (DNN) is an ANN consisting of five or more layers of algorithms connected and organised according to the meaningfulness of the data, and this enables improved predictions from data [13-16]. These layers store data from inputs and provide an output that is liable to change in an orderly manner once the AI system learns new features from the data [13-16]. DNNs are good in that they continuously improve as the size of the training data increases and doesn’t only work as a classifier but also as a feature extractor [13-17]. The differences in the DNN and simple neural network are illustrated in Figure 1.

Convolutional Neural Networks

**Figure 2:**
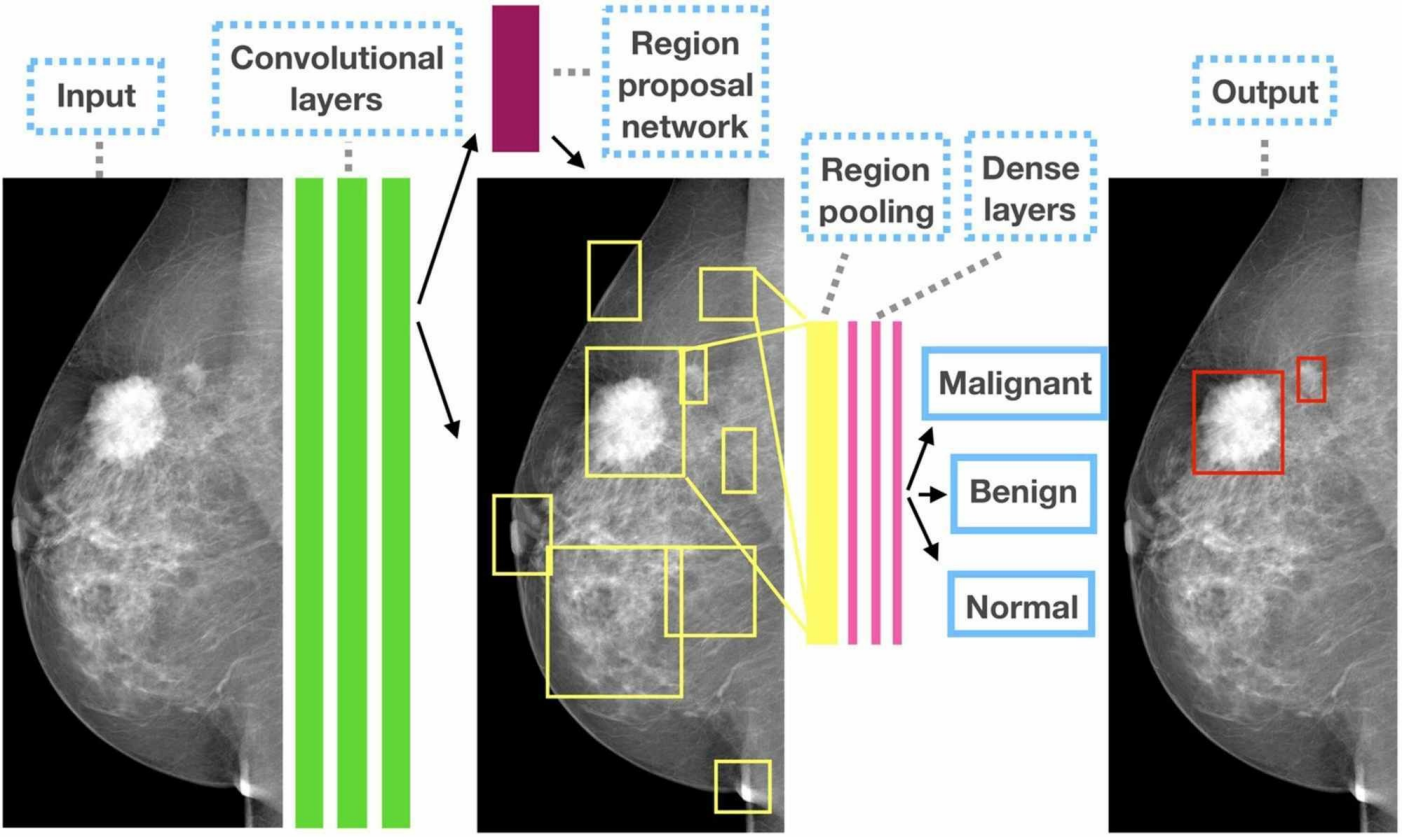
Schematic illustration of how a convolutional neural network extracts data and process it to give an output Photo credit: Ribli et al. [18].

DL is a typical network that takes one-dimensional inputs, while convolutional neural networks (CNNs) take two- or three-dimensional shaped data [15,18]. Convolution is a mathematical principle that is used to find repeated features in images [15,18]. CNNs consist of an input, an output, as well as many hidden layers that get useful information by convolving (filtering) the data. CNNs are the most commonly used AI tool in breast imaging [15,18]. Figure 2 shows how a CNN extracts data from an image and process the data to give a possible diagnosis.

Transfer Learning

Transfer learning is when knowledge obtained from a different experience is used on a separate but related job, for example, the use of an algorithm trained for non-medical visual recognition in feature extraction on mammograms [15]. Knowledge attained from regular photo image analysis is transferable to mammogram analysis [15].

Use of AI in breast imaging

The role of AI in breast imaging is not about finding whether machines are more ingenious than humans. Instead, AI is for expanding, sharpening, and relaxing the mind of the radiologist so that radiologists can do the same for their patients [19]. Following are the current uses and possible future uses of AI in breast radiology.

*Image Interpretation* 

Advances in AI and imaging technology led to an increase in the proposed applications of AI in breast radiology. A number of studies have explored the use of deep learning in mammogram interpretation. DL tried in making a diagnosis of breast pathology in a number of cases, such as differentiating benign from malignant breast masses, separating masses from micro-calcifications, distinguishing between tumor and healthy tissue, discrimination between benign, malignant, and healthy tissue and detect masses in mammogram images [20-24]. AI has potential use in density segmentation and risk calculation, classifying breast tissue into different densities, namely, scattered and uniformly dense breast density categories, image segmentation that is mapping the edges of a lesion, lesion identification, measurement, labeling, comparison with previous images, comparing images from both left and right breasts and also the craniocaudal and mediolateral-oblique view of each breast and breast anatomy classification in mammograms [25-28].

Radiomics 

Radiomics is extraction of large amounts of features from diagnostic images, using algorithms [29,30]. These features are known as radiomic features [29,30]. On a mammogram, radiomics extract vast amounts of features that a human eye cannot see and link these features with each other and other data [29,30]. The obtained data provides valuable information to radiologists and helps to predict prognosis, treatment response and many different potential uses such as differentiating benign and malignant breast tumors [30].

Imaging Banks

There are now vast amounts of data stored due to the continually increasing memory capacity of computers [29]. Overloaded picture archiving and communications system (PACS) is now familiar because we save raw images and lots of data generated from imaging [29]. AI can be used to store quantitative photos, and these stored images would be a handy tool to use as training data sets through which algorithms are trained [29].

Structured Reporting

AI can be used to make reporting standard by helping with the reporting template, assist with choice of vocabulary and recommend the most probable diagnosis [31].

Deciding Exam Priority Level

AI tools can also help the radiologist deciding exam priority using appropriateness criteria [15]. A clinical decision support (CDS) system helps referring clinicians in deciding the most relevant imaging procedure [32,33]. AI together with CDS, can make the process better and ultimately improve efficiency in the radiology department [29,33].

Quick Identification of Negative Studies

AI can be used to improve sensitivity by finding studies that are normal and leaving the rest for human readers [29,34]. This practice would be useful in high-volume sites or where double reporting is practiced [29,34]. Another way is to use AI to classify exams into normal and abnormal, and one radiologist will read those labelled as normal while the abnormal ones will go for double reading [29,34].

Clerical Work and Clinical Data Management

Physicians are now loaded with increasing amounts of administrative work, and this is among the leading causes of burnout. We can rope in AI applications in this area [34-37]. The ultimate goal expected from such ML-based systems is to assist healthcare workers cut documentation time and improve on report quality [34-37].

AI challenges

Acceptance of AI

In the early days of autopilot, pilots were reluctant to accept the technology, and this is the current situation now with radiologists fearing for their jobs [34-38]. However, technological advancements are not new to radiology; they were always part of the specialty, and technology is what drives radiology [34-38]. Bertalan Mesko has dubbed AI “the stethoscope of the 21st century” [36]. It was not easy for the early physicians to incorporate and trust the stethoscope as one of their best tools [36]. There is a need to sensitize people about AI through different channels to make the adoption of AI smooth [37]. We should not forget acceptance by patients as well. There is need to sensitize patients about AI. We also need consent from patients to use AI on image interpretation. Patients should be able to choose between AI and humans.

Training Data

Vast amounts of images are now available from the PACS, but the challenge arises in labeling the data for AI training [28-38]. Image labeling takes a lot of time and needs a lot of effort, and also, this process must be very robust [29-38]. Another problem comes with rare conditions; it is difficult to find enough images to train the algorithm so that the algorithm can identify them on its own in future [29-38]. Sometimes random variations on pictures can be seen by the program as a pathological lesion [29-38]. If input data used in training is from a different ethnic group, age group or different gender, it may give different results if given raw data from other diverse groups of people [15].

Medicolegal Issues

The question is if AI systems make an autonomous decision and make a mistake, who is responsible: radiologist, machine or builder of the device [38-43]? Physicians always take responsibility for the medical decisions made for patients. In case something went wrong, the programmers may not take responsibility, given that, the machines are continuously learning in ways not known by the developers. Recommendations provided by the tool may need to be ratified by a radiologist, who may agree or disagree with the software [38-43].

Is it the end for breast radiologists?

Geoffrey Hinton, a cognitive psychologist and computer scientist from Canada, was quoted saying, "if you work as a radiologist, you're like the coyote that's already over the edge of the cliff, but hasn't yet looked down so doesn't realize there's no ground underneath him. People should stop training radiologists now. It's just self-evident that within five years, deep learning is going to do better than radiologists; we've got plenty of radiologists already" [29]. Yes, in terms of image analysis and computing, AI has the potential to be more efficient than radiologists [29-45]. Radiologists cannot process millions of mammograms in any reasonable space of time, and this has led to some ideas that radiologists are going to be displaced by AI [29-45]. In the recent years, we have seen algorithms in various domains that are comparable or even better than humans in various radiological tasks, especially in breast imaging, which is why it is our main focus in this article [29-45]. Because of this success of DL in breast imaging, people started discussing the possibility of automating image interpretation and bury radiologists. These are far-fetched expectations; AI systems have their limitations discussed above. Also, it is crucial to know that AI is good at solving super-specific isolated problems [15-29]. In contrast, humans can understand different concepts, reason and put together vast amounts of information from various aspects and come up with an inclusive decision [29-45]. It is difficult to predict the future of AI on radiology. Still, many authors believe that AI will become part and parcel of the daily job of radiologists, making them more efficient [29-45]. AI will be valuable in performing routine tasks and help radiologists to concentrate on more useful jobs [38-45]. Radiologists will spend more time discussing with clinicians, in multi-disciplinary team meetings contributing to the long-term care of patients. More time will also be freed up and will enable them to communicate abnormal results in person or through a telephone call, and even quality enforcement, education, policy formulation and interventional procedures [29-42]. In general, when automation comes, jobs are not lost, but humans promote themselves to tasks needing a human touch [43].

Future roles of a radiologist

Since Roentgen, AI will be the biggest thing to come into radiology. Radiologists have a vital role to play for the good of both AI and radiologists [29-44]. They are an essential piece of the puzzle for several challenges AI is facing now, such as the production of training images [29-47]. There is a need for many labelled images provided by experienced radiologists and creating these datasets is difficult and time-consuming [29-45]. However, even if necessary, the radiologist's role is not only image labeling. Radiologists should be involved in directing programmers to areas where AI methods are needed most because they are the end-users, and they know where they need help [40-47]. There is a need to increase partnership with AI developers in the development of applications to be used in breast radiology [46]. These professionals should become part and parcel of radiological departments, creating a “multidisciplinary AI team” that will ensure the adequacy of patient safety standards. In this way, radiologists can take responsibility for legal liability [45,46]. There is also a need for the development of rigorous evaluation criteria of applications before they are licensed for use and radiologists should take this responsibility [29-47].

## Conclusions

Indeed, AI will change radiology, and it is not too early to incorporate it into your workplace. While there are even more grey areas that need clarification, our opinion is that AI will not replace radiologists. Still, those who incorporate AI in their daily work will probably be better off than those who don't. Every radiologist must prepare for a future of working with machines more. We predict a future where radiologists will continue to make strides and draw many benefits from the use of more sophisticated AI systems over the next 10 years. If you are interested in breast radiology, from medical students to radiologists, anticipate a more satisfying and rewarding career, especially if you have skills in programming. AI knowledge and data science must be a part of the medical school and radiology trainees’ curriculum. Finally, it is important to say our patients, not AI, should be central to every decision that we take as a professional.
